# Resection of a Pediatric Thalamic Juvenile Pilocytic Astrocytoma with Whole Brain Tractography

**DOI:** 10.7759/cureus.1768

**Published:** 2017-10-11

**Authors:** Howard L Weiner, Dimitris G Placantonakis

**Affiliations:** 1 Division of Pediatric Neurosurgery, Department of Surgery, Texas Children’s Hospital; 2 Neurosurgery, NYU School of Medicine

**Keywords:** whole brain tractography, juvenile pilocytic astrocytoma, tubular retractor, thalamus

## Abstract

The resection of deep-seated brain tumors has been associated with morbidity due to injury to critical neural structures during the approach. Recent technological advancements in navigation and stereotaxy, surgical planning, brain tractography and minimal-access brain ports present the opportunity to overcome such limitations. Here, we present the case of a pediatric patient with a left thalamic/midbrain juvenile pilocytic astrocytoma (JPA). The tumor displaced the corticospinal fibers posteriorly and resulted in hemiparesis. Using whole brain tractography to plan a corridor for the approach, neuronavigation, a tubular retractor and an exoscope for visualization, we obtained gross total resection of the tumor, while minimizing injury to white matter bundles, including the corticospinal fibers. We propose that surgical planning with whole brain tractography is essential for reducing morbidity while accessing deep-lying brain lesions via retractor tubes, by means of sparing critical fiber tracts.

## Introduction

Deep-seated intracranial tumors have always presented a challenge to neurosurgeons because surgical approaches for their resection have been associated with neurologic deficits and morbidity. Due to these risks, centrally located neoplasms in the thalamus, basal ganglia, and subcortical white matter have often been deemed not resectable.

As early as 30 years ago, pioneers in our field started making use of imaging and stereotaxy to access and resect deep-lying tumors. Kelly was one of the first surgeons to describe the use of image-guided stereotaxy and tubular retractors to access deep intracranial tumors [1-2]. The premise of using tubes to create a surgical corridor lies in the attempt to preserve the subcortical white matter in the approach. While brain retraction with devices like the Greenberg retractor system is thought to disrupt and injure neural tissue and tubes are believed to reduce such injury by displacing tissue. The concept of tubular retraction recently gained traction again with two commercially available designs. As a result, several case series of minimal access surgery through tubes for the resection of deep-lying neoplasms or hemorrhages have appeared in the neurosurgical literature in recent years [3-7].

It is important to note that while tubular retractors reduce white matter injury, such surgical corridors do show some evidence of damage, as shown by diffusion restriction studies [8]. It is therefore important to plan the trajectory of the tube to the lesion so that the most critical white matter bundles are spared. Whole brain tractography is essential for sparing white matter tracts during the planning of the surgical corridor. Some commercially available planning/neuronavigation platforms now offer integration of whole brain tractography into conventional magnetic resonance imaging (MRI) to facilitate such planning. One such platform is BrightMatter planning software (Synaptive Medical, Toronto, Canada). The planning software uses whole brain tractography to help identify surgical corridors that do not disrupt critical fiber bundles but rather displace them.

In this report, we describe the use of BrightMatter planning software and a cylindrical tubular retractor (BrainPath, Nico, Indianapolis, USA) to access and completely resect a thalamic/midbrain juvenile pilocytic astrocytoma (JPA) in a pediatric patient. We also discuss nuances of the system that make it ideal for planning such surgical corridors and contemplate areas of future investigation.

## Case presentation

Initial presentation

The patient was a 10-year-old left-handed male who presented with progressive right upper extremity paresis for over a year. The right lower extremity showed only minor weakness. At the time of presentation, the right hand had severe difficulty with skilled movements. Physical examination showed atrophy of the right hand and to a lesser extent muscle in the right lower extremity. Neurologic examination was notable for 3+/ 5 strength and poor fine motor skills in the distal right upper extremity. There were 3+/4 hyperreflexia and a positive Babinski reflex on the right. The patients' cognition was within normal.

**Figure 1:**
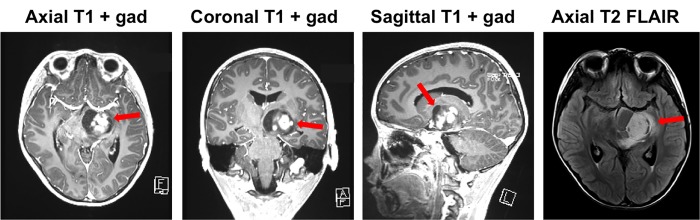
Preoperative magnetic resonance imaging (MRI). Preoperative MRI showing the tumor (red arrow) in the left thalamus/midbrain. The FLAIR (fluid-attenuated inversion recovery) image demonstrates mild peritumoral edema, gad: gadolinium.

The MRI indicated a cystic lesion in the left anterior thalamus and midbrain with an enhancing nodular component (Figure 1). There were mild surrounding vasogenic edema and mass effect on the third ventricle. The radiographic appearance was suggestive of JPA.

Surgical planning

**Figure 2:**
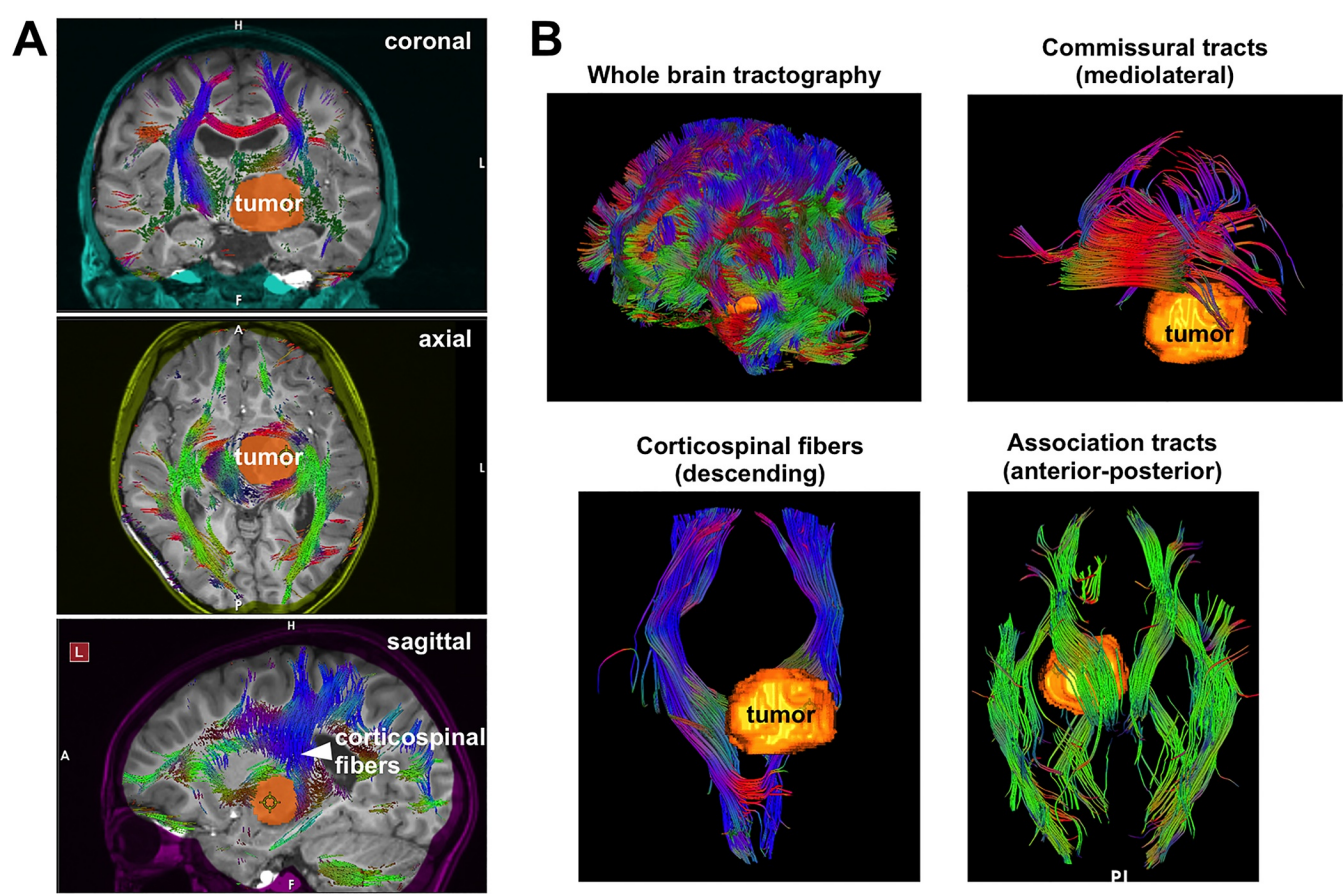
The spatial relationship of the tumor to white matter tracts. Whole brain tractography was used to assess the spatial relationship of the subcortical white matter tracts to the tumor. A: Two-dimensional (2D) views of the tumor and white matter tracts are shown on the coronal, axial and sagittal planes. Note: The corticospinal fibers are draped over the posterior aspect of the tumor. B: Whole brain three-dimensional (3D) tractography demonstrates these relationships in 3D space. In both (A) and (B), the tumor has been “painted” orange for easy identification.

**Figure 3:**
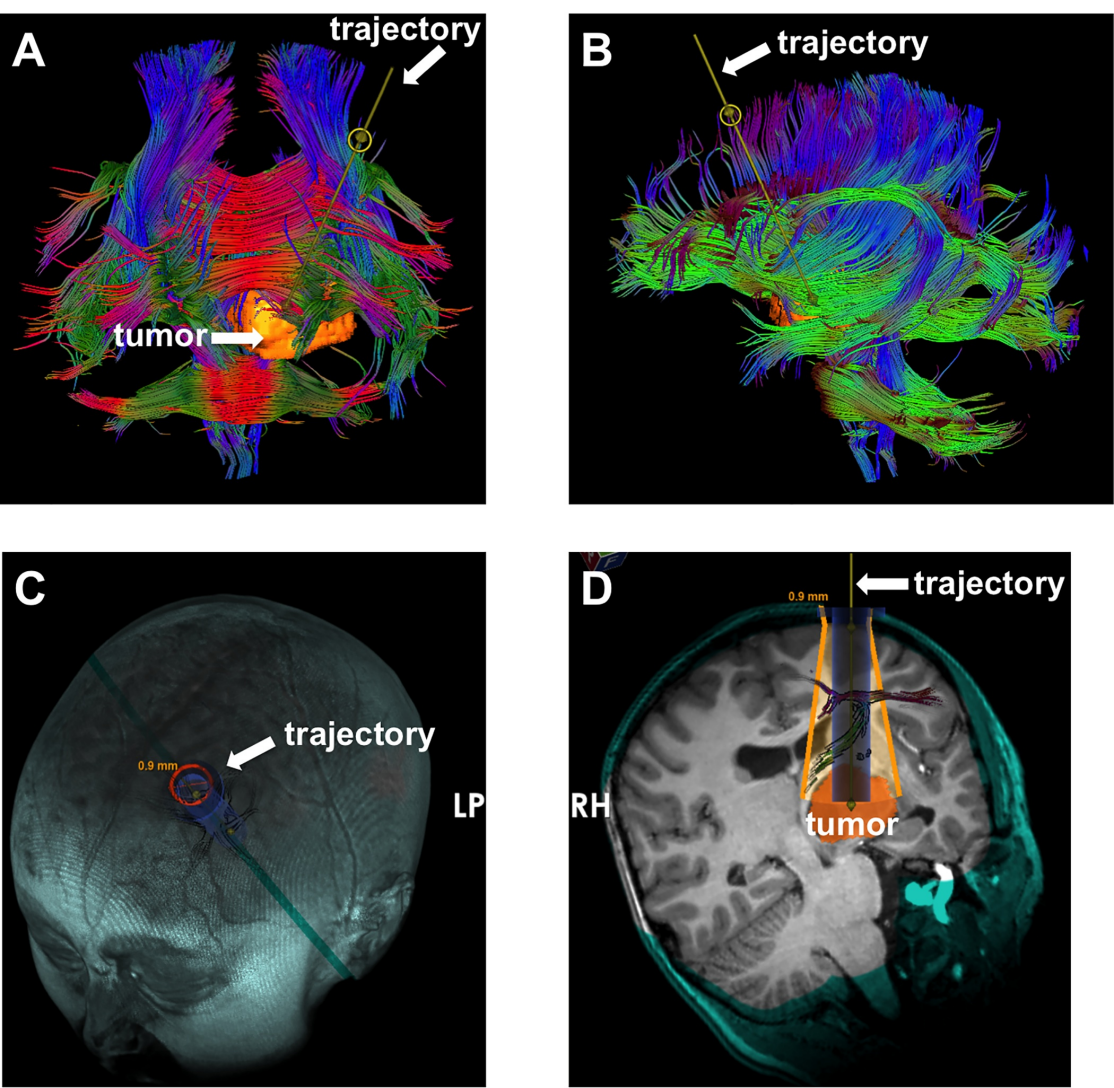
Planning of the surgical corridor. A and B: Projections of the planned trajectory in relation to subcortical white matter tracts. C: Three dimensional (3D) surface rendering of the planned entry site of the tubular retractor on the scalp. D: Inline view of the planned trajectory and intersecting fiber tracts. Note: The software indicates the anticipated range of the movement of the retractor in order to capture the entire tumor diameter.

In preparation for surgery, he underwent diffusion tensor imaging (DTI). Whole brain tractography using Synaptive’s BrightMatter software indicated that the corticospinal fibers were displaced to the posterior margin of the tumor (Figure 2). We planned a trans-sulcal approach to the lesion with a 7.5 cm long 13.5 mm wide cylindrical retractor tube (BrainPath, Nico, Indianapolis, USA). Given the location in the anterior thalamus, we optimized a trajectory with an entry point in the left superior frontal sulcus in the pre-coronal space and a course through the anterior limb of the internal capsule (Figure 3). Whole brain tractography allowed us to finesse the surgical corridor in order to not directly traverse longitudinal association fibers within the left frontal lobe (Figure 3).

Procedure

Under general anesthesia, the patient’s head was placed in a Sugita frame. Using the BrainLab neuronavigation system, we identified the projection of the pre-planned trajectory on the scalp of the left frontal convexity and designed an S-shaped incision around it. We generated a 4 cm circular craniotomy centered on our trajectory and incised the dura in cruciate fashion, each incision about 2 cm in length. We performed a small corticotomy within the superior frontal sulcus and cannulated the brain with the tubular retractor under neuronavigation guidance.

Upon reaching our target, we decanted the BrainPath tube and brought in an exoscope (Vitom, Karl Storz, El Segundo, California) for visualization. We removed the tumor tissue and decompressed the cystic component. During the resection of the tumor, there was a mild decline in the amplitude of motor evoked potentials (MEPs).

Postoperative course

**Figure 4:**
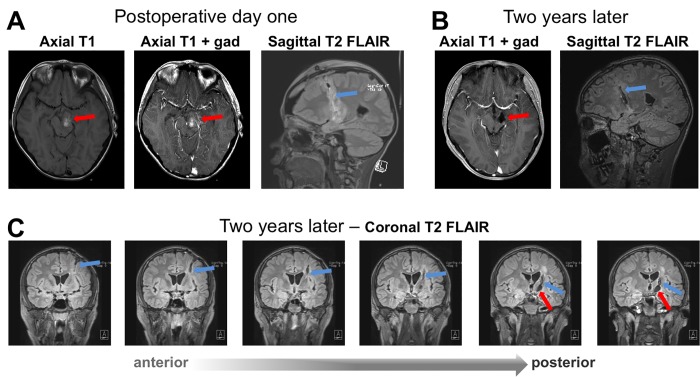
Postoperative magnetic resonance imaging (MRI). Postoperative MRIs obtained on day one (A) and two years (B and C) after surgery demonstrate gross total resection of the juvenile pilocytic astrocytoma (JPA) and no recurrence. The panel in (C) represents serial thin-cut 1 mm T2 fluid-attenuated inversion recovery (FLAIR) images on the coronal plane that capture the surgical corridor two years after the procedure. Note: The trajectory traverses the anterior limb of the internal capsule as planned and is surrounded by only small amounts of FLAIR hyperintensity. This suggests a limited gliosis due to injury during the approach. The red arrow: the surgical cavity; blue arrow: trajectory.

Postoperative MRI indicated gross total resection of the tumor (Figure 4A). The pathology was consistent with JPA with a MIB-1 index of 2%. There was a transient postoperative worsening of the right hemiparesis, as well as a new left oculomotor palsy. These deficits improved significantly with rehabilitation.

His most recent MRI two years after surgery shows no recurrence (Figure 4B). Serial thin cut T2 fluid-attenuated inversion recovery (FLAIR) images along the surgical corridor showed minimal gliosis (Figure 4C). Neurologically, there is near complete recovery of his hemiparesis and he has started to participate in sports activities. The oculomotor palsy was largely corrected. He is being followed by ophthalmology for the oculomotor palsy and corrective surgery may be considered in the future for any residual deficit. From the cognitive point of view, he was living a normal life and was excelling in school.

## Discussion

Thalamic JPAs and other centrally located neoplasms present a surgical challenge. Several surgical corridors have been proposed for approaching such lesions. However, these approaches are often associated with morbidity. The advancements in DTI imaging have allowed the visualization of critical fiber bundles in the subcortical white matter. Visualizing the spatial relationship of these tracts in relation to the tumor is the key to surgical planning and in reducing the risk of neurologic morbidity.

While the combination of advanced imaging and tubular retractors provide the basic technology required to access central intracranial neoplasms safely, refining the surgical corridor had not been possible until the advent of the whole brain tractography planning platforms. An example of this technology is Synaptive’s BrightMatter software. The BrightMatter is a trajectory-focused imaging platform that displays white matter fibers on planes orthogonal to the planned trajectory, allowing for optimization of the surgical corridor. The ideal trajectory should not traverse fiber bundles, but rather use corridors between tracts to displace but not impale them. The surgeons have traditionally paid attention to fiber tracts whose injury results in neurologic deficits: corticospinal fibers, arcuate fasciculus, optic radiations. However, the whole brain tractography allows sparing of other bundles, such as association fibers in longitudinal fasciculi, which serve higher-order cognitive functions.

This exciting new technology is certain to evolve. Automated identification and naming of fiber bundles on the basis of consensus white matter tract maps is underway. Fiber tracking of cranial nerves, for example, the oculomotor nerve, in this case, will certainly afford greater surgical precision. Integration of trajectory-based planning platforms with other imaging modalities and navigation systems will be necessary to provide surgeons with all necessary information to carry out safe procedures. Intraoperative visualization of fiber tracts superimposed on the microscope or exoscope images will undoubtedly help bring white matter fiber bundle anatomy into surgery.

The next few years will also help answer another important question related to deep-seated intracranial neoplasms: Is resection the best way to treat them?. Laser ablation of tumors certainly provides an alternative to excision, with potentially less morbidity but with the disadvantage of the persistent mass effect of the tumor, at least until the coagulum is cleared by the immune system. As collective experience grows with these new technologies, we hope to be able to answer questions related to the relative neurologic safety and oncologic efficacy of minimally invasive tumor excision vs. laser ablation.

## Conclusions

Advanced surgical planning with whole brain tractography allows sparing of major white matter tracts during cannulation of the brain with tubular retractors toward resection of deep-seated tumors. While there is room for further improvement in imaging, navigation, surgical planning, and intraoperative visualization, these novel technologies allow relatively safe resections of centrally located neoplasms that would otherwise be deemed as high-risk procedures. The presented case illustrates the potential of these techniques combined to produce an excellent clinical outcome after surgery for a thalamic/midbrain benign tumor.
